# (4-Chloro­phen­yl)methanaminium chloride hemihydrate

**DOI:** 10.1107/S1600536810021100

**Published:** 2010-06-16

**Authors:** Sofiane Souissi, Wajda Smirani Sta, Salem S. Al-Deyab, Mohamed Rzaigui

**Affiliations:** a Laboratoire de Chimie des Matériaux, Faculté des Sciences de Bizerte, 7021 Zarzouna Bizerte, Tunisia; b Petrochemical Research Chair, College of Science, King Saud University, Riyadh, Saudi Arabia

## Abstract

In the title hydrated salt, C_7_H_9_ClN^+^·Cl^−^·0.5H_2_O, the water O atom lies on a crystallographic twofold axis. In the crystal, the monoprotonated 4-chloro­benzyl­ammonium cation forms N—H⋯Cl and N—H⋯O hydrogen bonds and the water mol­ecule forms O—H⋯Cl hydrogen bonds, generating layers lying parallel to the *bc* plane.

## Related literature

For the properties of benzyl­amines, see: Markwardt *et al.* (2005 ▶). For a related structure, see: Dhaouadi *et al.* (2008 ▶). 
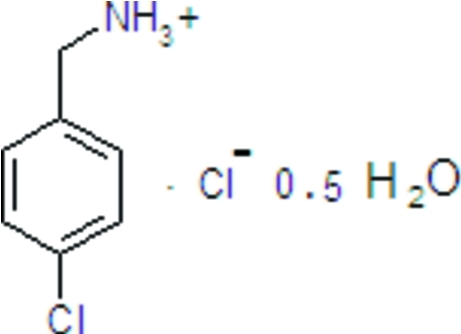


## Experimental

### 

#### Crystal data

C_7_H_9_ClN^+^·Cl^−^·0.5H_2_O
*M* *_r_* = 187.06
Monoclinic, 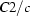

*a* = 30.462 (2) Å
*b* = 4.890 (3) Å
*c* = 11.738 (2) Å
β = 99.97 (3)°
*V* = 1722.1 (11) Å^3^
*Z* = 8
Ag *K*α radiation
λ = 0.56085 Å
μ = 0.35 mm^−1^
*T* = 293 K
0.30 × 0.25 × 0.20 mm

#### Data collection

Enraf–Nonius TurboCAD-4 diffractometer
5908 measured reflections
4207 independent reflections
2217 reflections with *I* > 2σ(*I*)
*R*_int_ = 0.031
2 standard reflections every 120 min  intensity decay: 5%

#### Refinement

*R*[*F* ^2^ > 2σ(*F* ^2^)] = 0.048
*wR*(*F* ^2^) = 0.130
*S* = 1.00
4207 reflections
101 parameters
H atoms treated by a mixture of independent and constrained refinement
Δρ_max_ = 0.34 e Å^−3^
Δρ_min_ = −0.32 e Å^−3^

### 

Data collection: *CAD-4 EXPRESS* (Enraf–Nonius, 1994 ▶); cell refinement: *CAD-4 EXPRESS*; data reduction: *XCAD4* (Harms & Wocadlo, 1995 ▶); program(s) used to solve structure: *SHELXS97* (Sheldrick, 2008 ▶); program(s) used to refine structure: *SHELXL97* (Sheldrick, 2008 ▶); molecular graphics: *ORTEP-3* (Farrugia, 1997 ▶); software used to prepare material for publication: *WinGX* (Farrugia, 1999 ▶).

## Supplementary Material

Crystal structure: contains datablocks I, global. DOI: 10.1107/S1600536810021100/hb5481sup1.cif

Structure factors: contains datablocks I. DOI: 10.1107/S1600536810021100/hb5481Isup2.hkl

Additional supplementary materials:  crystallographic information; 3D view; checkCIF report

## Figures and Tables

**Table 1:** Hydrogen-bond geometry (Å, °)

*D*—H⋯*A*	*D*—H	H⋯*A*	*D*⋯*A*	*D*—H⋯*A*
N—H0*A*⋯Cl1^i^	0.89	2.60	3.2930 (19)	136
N—H0*A*⋯Cl1^ii^	0.89	2.78	3.417 (2)	130
N—H0*B*⋯O	0.89	2.04	2.866 (2)	155
N—H0*C*⋯Cl1^iii^	0.89	2.26	3.144 (2)	175
O—H1⋯Cl1	0.85 (3)	2.28 (3)	3.1230 (18)	171 (3)

Symmetry codes: (i) 
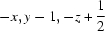
; (ii) 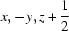; (iii) 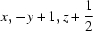.

**Fig. 1.**
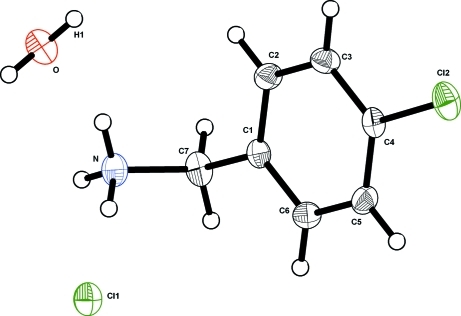
View of (I) with displacement ellipsoids for non-H atoms are drawn at the 30% probability level.

**Fig. 2.**
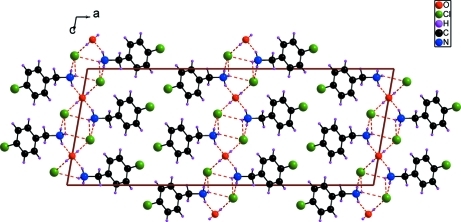
A view of the packing of (I) along the b axis.

## supplementary crystallographic information

### Comment

Derivatives of benzylamine were found to be competitive inhibitors of the proteolytic enzymes trypsin, plasmin, and thrombin. So, the 4-chlorobenzylamine is a strong thrombin inhibitor but only of low effectiveness against trypsin and plasmin for the hydrolysis of N-α-benzoyl catalyzed by these three enzymes. Relations between the chemical structure and the activity against trypsin, plasmin and thrombin were deduced by comparing the inhibitor constants (Markwardt, F. *et al.*, 2005). In this work, we report the crystal structure of the title compound (I). As shown in (Fig.1), the asymmetric unit of (I) is built up from one 4-chlorobenzylammonium cation, one chloride anion and one water molecule. The Cl^-^ anions, water molecules and R—NH_3_^+^ groups are lineked *via* O—H···Cl, N—H···O and N—H···Cl hydrogen bonds and ionic interactions, so as to built inorganic layers spreading around the (b,c) planes. The 4-chlorobenzylammonium cations are anchored onto the successive inorganic layers *via* hydrogen bonds and electrostatic interactions, to composite their negative charges.

The examination of the organic cation shows that the values of N—C, C—C, C—Cl distances and N—C—C, C—C—C, C—C—Cl angles range from 1.376 (3) to 1.736 (3) Å and 115.72 (19) to 122.80 (19)°, respectively. These values show no significant difference from those obtained in other organic materials associated with the same organic groups (Dhaouadi, H. *et al.*, 2008).

In this structure, the water molecules play a very important role in the cohesion of the various groups. It participates with the organic cations and chloride anions in the H-bonding scheme of N—H···O and O—H···Cl interactions in the crystal structure. The four hydrogen bonds are relatively weak, and their donor acceptor distances vary from 2.866 (2) to 3.417 (3) Å. Thus, these different interactions (hydrogen bonds, Van der Waals, and electrostatic) form a stable three-dimensional network.

### Experimental

An ethanolic solution of 4-chlorobenzylamine (10 mmol, in 10 ml) was added, with stirring, to 20 ml of an aqueous HCl solution (0.5*M*) at room temperature. Colourless blocks of (I) were obtained on slow evaporation of the solvent.

### Refinement

All H atoms were positioned geometrically and treated as riding on their parent atoms, [N–H = 0.89, C–H =0.96 Å (CH_3 _) with with *U*_iso_(H) = 1.5Ueq and C–H =0.96 Å (Ar–H), with *U*_iso_(H) = 1.5Ueq], but those attached to oxygen atom are located in a difference map

### Crystal data

**Table:**

C_7_H_9_ClN^+^·Cl^−^·0.5H_2_O	*F*(000) = 776
*M**_r_* = 187.06	*D*_x_ = 1.443 Mg m^−^^3^
Monoclinic, *C*2/*c*	Ag *K*α radiation, λ = 0.56085 Å
Hall symbol: -C 2yc	Cell parameters from 25 reflections
*a* = 30.462 (2) Å	θ = 9–11°
*b* = 4.890 (3) Å	µ = 0.35 mm^−^^1^
*c* = 11.738 (2) Å	*T* = 293 K
β = 99.97 (3)°	Block, colourless
*V* = 1722.1 (11) Å^3^	0.30 × 0.25 × 0.20 mm
*Z* = 8	

### Data collection

**Table:**

Enraf–Nonius TurboCAD-4 diffractometer	*R*_int_ = 0.031
Radiation source: fine-focus sealed tube	θ_max_ = 28.0°, θ_min_ = 2.3°
graphite	*h* = −50→50
non–profiled ω scans	*k* = 0→8
5908 measured reflections	*l* = −5→19
4207 independent reflections	2 standard reflections every 120 min
2217 reflections with *I* > 2σ(*I*)	intensity decay: 5%

### Refinement

**Table:**

Refinement on *F*^2^	Secondary atom site location: difference Fourier map
Least-squares matrix: full	Hydrogen site location: inferred from neighbouring sites
*R*[*F*^2^ > 2σ(*F*^2^)] = 0.048	H atoms treated by a mixture of independent and constrained refinement
*wR*(*F*^2^) = 0.130	*w* = 1/[σ^2^(*F*_o_^2^) + (0.0585*P*)^2^ + 0.2911*P*] where *P* = (*F*_o_^2^ + 2*F*_c_^2^)/3
*S* = 1.00	(Δ/σ)_max_ = 0.001
4207 reflections	Δρ_max_ = 0.34 e Å^−^^3^
101 parameters	Δρ_min_ = −0.32 e Å^−^^3^
0 restraints	Extinction correction: *SHELXL97* (Sheldrick, 2008), Fc^*^=kFc[1+0.001xFc^2^λ^3^/sin(2θ)]^-1/4^
Primary atom site location: structure-invariant direct methods	Extinction coefficient: 0.0080 (12)

### Special details

**Table:**

Geometry. All e.s.d.'s (except the e.s.d. in the dihedral angle between two l.s. planes) are estimated using the full covariance matrix. The cell e.s.d.'s are taken into account individually in the estimation of e.s.d.'s in distances, angles and torsion angles; correlations between e.s.d.'s in cell parameters are only used when they are defined by crystal symmetry. An approximate (isotropic) treatment of cell e.s.d.'s is used for estimating e.s.d.'s involving l.s. planes.
Refinement. Refinement of *F*^2^ against ALL reflections. The weighted *R*-factor *wR* and goodness of fit *S* are based on *F*^2^, conventional *R*-factors *R* are based on *F*, with *F* set to zero for negative *F*^2^. The threshold expression of *F*^2^ > σ(*F*^2^) is used only for calculating *R*-factors(gt) *etc*. and is not relevant to the choice of reflections for refinement. *R*-factors based on *F*^2^ are statistically about twice as large as those based on *F*, and *R*- factors based on ALL data will be even larger.

### Fractional atomic coordinates and isotropic or equivalent isotropic displacement parameters (Å^2^)

**Table:**

	*x*	*y*	*z*	*U*_iso_*/*U*_eq_	
Cl1	0.049348 (13)	0.63727 (9)	0.11132 (4)	0.04257 (13)	
O	0.0000	0.2285 (4)	0.2500	0.0494 (5)	
H1	0.0151 (8)	0.323 (5)	0.210 (2)	0.089 (9)*	
C1	0.12851 (4)	0.0280 (3)	0.40289 (14)	0.0313 (3)	
C2	0.12629 (5)	0.1317 (3)	0.29253 (14)	0.0364 (3)	
H2	0.1048	0.0661	0.2328	0.044*	
C3	0.15582 (5)	0.3328 (3)	0.26977 (14)	0.0371 (3)	
H3	0.1538	0.4046	0.1957	0.045*	
C4	0.18814 (4)	0.4244 (3)	0.35851 (14)	0.0329 (3)	
C5	0.19059 (5)	0.3278 (4)	0.46938 (15)	0.0386 (4)	
H5	0.2121	0.3947	0.5289	0.046*	
C6	0.16065 (5)	0.1293 (4)	0.49129 (14)	0.0380 (3)	
H6	0.1621	0.0631	0.5661	0.046*	
C7	0.09748 (5)	−0.1977 (3)	0.42512 (18)	0.0399 (4)	
H7A	0.0966	−0.3362	0.3656	0.048*	
H7B	0.1090	−0.2824	0.4990	0.048*	
Cl2	0.225682 (14)	0.66901 (9)	0.32754 (5)	0.04948 (15)	
N	0.05166 (4)	−0.0999 (3)	0.42641 (13)	0.0404 (3)	
H0A	0.0347	−0.2400	0.4401	0.061*	
H0B	0.0406	−0.0261	0.3582	0.061*	
H0C	0.0521	0.0251	0.4817	0.061*	

### Atomic displacement parameters (Å^2^)

**Table:**

	*U*^11^	*U*^22^	*U*^33^	*U*^12^	*U*^13^	*U*^23^
Cl1	0.0390 (2)	0.0365 (2)	0.0527 (3)	0.00140 (16)	0.00925 (17)	0.00195 (19)
O	0.0523 (11)	0.0428 (10)	0.0574 (12)	0.000	0.0215 (9)	0.000
C1	0.0289 (6)	0.0260 (6)	0.0403 (8)	0.0011 (5)	0.0097 (6)	−0.0002 (6)
C2	0.0372 (7)	0.0376 (8)	0.0333 (8)	−0.0070 (6)	0.0033 (6)	−0.0044 (7)
C3	0.0441 (8)	0.0367 (8)	0.0314 (8)	−0.0060 (7)	0.0086 (6)	0.0008 (7)
C4	0.0281 (6)	0.0281 (6)	0.0444 (9)	−0.0017 (5)	0.0117 (6)	−0.0042 (6)
C5	0.0318 (7)	0.0436 (9)	0.0388 (9)	−0.0035 (6)	0.0011 (6)	−0.0064 (7)
C6	0.0388 (7)	0.0407 (8)	0.0342 (8)	0.0015 (7)	0.0057 (6)	0.0049 (7)
C7	0.0384 (7)	0.0263 (7)	0.0577 (11)	0.0010 (6)	0.0153 (7)	0.0043 (7)
Cl2	0.0432 (2)	0.0391 (2)	0.0709 (3)	−0.01276 (17)	0.0229 (2)	−0.0055 (2)
N	0.0339 (6)	0.0355 (7)	0.0529 (9)	−0.0069 (5)	0.0101 (6)	0.0005 (6)

### Geometric parameters (Å, °)

**Table:**

O—H1	0.84 (2)	C5—C6	1.386 (2)
C1—C2	1.382 (2)	C5—H5	0.9300
C1—C6	1.389 (2)	C6—H6	0.9300
C1—C7	1.505 (2)	C7—N	1.4779 (19)
C2—C3	1.389 (2)	C7—H7A	0.9700
C2—H2	0.9300	C7—H7B	0.9700
C3—C4	1.379 (2)	N—H0A	0.8900
C3—H3	0.9300	N—H0B	0.8900
C4—C5	1.374 (2)	N—H0C	0.8900
C4—Cl2	1.7361 (15)		
			
C2—C1—C6	118.89 (14)	C5—C6—C1	120.84 (15)
C2—C1—C7	120.10 (15)	C5—C6—H6	119.6
C6—C1—C7	120.98 (15)	C1—C6—H6	119.6
C1—C2—C3	120.78 (15)	N—C7—C1	112.77 (13)
C1—C2—H2	119.6	N—C7—H7A	109.0
C3—C2—H2	119.6	C1—C7—H7A	109.0
C4—C3—C2	119.10 (15)	N—C7—H7B	109.0
C4—C3—H3	120.5	C1—C7—H7B	109.0
C2—C3—H3	120.5	H7A—C7—H7B	107.8
C5—C4—C3	121.21 (14)	C7—N—H0A	109.5
C5—C4—Cl2	120.36 (12)	C7—N—H0B	109.5
C3—C4—Cl2	118.42 (13)	H0A—N—H0B	109.5
C4—C5—C6	119.14 (14)	C7—N—H0C	109.5
C4—C5—H5	120.4	H0A—N—H0C	109.5
C6—C5—H5	120.4	H0B—N—H0C	109.5

### Hydrogen-bond geometry (Å, °)

**Table:**

*D*—H···*A*	*D*—H	H···*A*	*D*···*A*	*D*—H···*A*
N—H0A···Cl1^i^	0.89	2.60	3.2930 (19)	136
N—H0A···Cl1^ii^	0.89	2.78	3.417 (2)	130
N—H0B···O	0.89	2.04	2.866 (2)	155
N—H0C···Cl1^iii^	0.89	2.26	3.144 (2)	175
O—H1···Cl1	0.85 (3)	2.28 (3)	3.1230 (18)	171 (3)

Symmetry codes: (i) −*x*, *y*−1, −*z*+1/2; (ii) *x*, −*y*, *z*+1/2;  (iii) *x*, −*y*+1, *z*+1/2.
